# The REDUCE metagram: a comprehensive prediction tool for determining the utility of dutasteride chemoprevention in men at risk for prostate cancer

**DOI:** 10.3389/fonc.2012.00138

**Published:** 2012-10-11

**Authors:** Carvell T. Nguyen, Brandon Isariyawongse, Changhong Yu, Michael W. Kattan

**Affiliations:** ^1^Department of Urology, Glickman Urological and Kidney Institute, Cleveland Clinic FoundationCleveland, OH, USA; ^2^Department of Quantitative Health Sciences, Cleveland Clinic FoundationCleveland, OH, USA

**Keywords:** prostatic neoplasms, nomogram, chemoprevention, prediction

## Abstract

**Introduction**: 5-alpha reductase inhibitors can reduce the risk of prostate cancer (PCa) but can be associated with significant side effects. A library of nomograms which predict the risk of clinical endpoints relevant to dutasteride treatment may help determine if chemoprevention is suited to the individual patient. **Methods**: Data from the REDUCE trial was used to identify predictive factors for 9 endpoints relevant to dutasteride treatment. Using the treatment and placebo groups from the biopsy cohort, Cox proportional hazards (PH) and competing risks regression (CRR) models were used to build 18 nomograms, whose predictive ability was measured by concordance index (CI) and calibration plots. **Results**: A total of 18 nomograms assessing the risks of cancer, high grade cancer, high grade prostatic intraepithelial neoplasia (HGPIN), atypical small acinar proliferation (ASAP), erectile dysfunction (ED), acute urinary retention (AUR), gynecomastia, urinary tract infection (UTI) and BPH-related surgery either on or off dutasteride were created. The nomograms for cancer, high grade cancer, ED, AUR, and BPH-related surgery demonstrated good discrimination and calibration while those for gynecomastia, UTI, HGPIN, and ASAP predicted no better than random chance. **Conclusions**: To aid patients in determining whether the benefits of dutasteride use outweigh the risks, we have developed a comprehensive metagram that can generate individualized risks of 9 outcomes relevant to men considering chemoprevention. Better models based on more predictive markers are needed for some of the endpoints but the current metagram demonstrates potential as a tool for patient counseling and decision-making that is accessible, intuitive, and clinically relevant.

## Introduction

Contemporary management of prostate cancer (PCa) has revolved around the early detection of disease achieved through prostate specific antigen (PSA) screening. This paradigm has led to favorable changes in the epidemiology of PCa, including a downward stage migration, increased rates of cure with definitive treatment, and a reduction in cancer-specific mortality (Catalona et al., 1993; Horner et al., 2009). However, PCa remains a disease that can inflict significant morbidity and mortality; indeed, it is still the second leading cause of cancer death in American men behind lung cancer in 2010 (National Cancer Institute, 2011). On the other hand, there is evidence that widespread PSA testing has led to an overdiagnosis and overtreatment of clinically insignificant disease, unnecessarily exposing men to treatment-related morbidity as well as incurring significant healthcare costs (Bill-Axelson et al., 2005; Welch and Albertsen, 2009). As a result, there has been growing interest in reducing a man's risk of developing PCa through chemoprevention and potentially avoiding these problems altogether.

In the last several years, data from studies assessing the effects of 5-alpha reductase inhibitors (5-ARI) have demonstrated significant benefits in the form of a 23 to 25% reduction in cancer risk as well as improvement of benign prostatic hyperplasia (BPH) related urinary symptoms (Thompson et al., 2003; Andriole et al., 2010). These findings have suggested a role for such drugs as chemoprevention in men at risk for developing PCa. However, treatment with 5-ARIs can be associated with significant side effects that may adversely impact quality of life and lead to discontinuation of the drug. For example, data from the Reduction by Dutasteride of Prostate Cancer Events (REDUCE) trial showed a significantly higher risk of sexual side effects, including loss of libido and erectile dysfunction (ED), as well as an increased incidence of gynecomastia and cardiac failure.

Therefore, the decision to initiate chemoprevention should not be taken lightly nor should it be applied indiscriminately to all men. With multiple clinical endpoints to consider, the process of calculating an individual man's risk-to-benefit ratio regarding 5-ARI treatment can be difficult for both physician and patient in the absence of a formalized system to predict risk. We have previously proposed a novel prediction tool, dubbed the “metagram,” that can facilitate decision-making by obviating physicians and patients from having to predict outcomes themselves or make complex calculations (Nguyen and Kattan, 2009). Such a comprehensive prediction tool would incorporate nomograms that can generate individualized predictions of all outcomes relevant to the clinical question and present the data in a manner that is easy to interpret.

Using data from the REDUCE trial that assessed the effects of dutasteride in men at high risk of PCa, we have constructed nomograms that predict the risks of nine clinically relevant endpoints in the absence or presence of dutasteride treatment. The nomograms have been incorporated into an online metagram program that can generate personalized predictions of the potential consequences of dutasteride treatment. Armed with such data, the individual patient can then make an informed decision regarding whether chemoprevention with a 5-ARI is right for him.

## Materials and methods

Data from 6729 patients from the REDUCE trial who had at least one biopsy or prostate surgery were included in this study (Andriole et al., 2010). This cohort was split into two sub-groups: (1) patients who received dutasteride (*N* = 3305) and (2) patients who received placebo (*N* = 3424).

Endpoints related to pathology, BPH, and drug-related side effects were studied (Table 1). The pathological endpoints included PCa, high grade prostate cancer (HGPCa) that was defined as Gleason score sum ≥7, high grade prostatic intraepithelial neoplasia (HGPIN), and atypical small acinar proliferation (ASAP). In this study, HGPIN was counted as an independent endpoint only if there was no previous or concurrent ASAP or PCa. Similarly, ASAP was counted only in the absence of PCa. The endpoints related to BPH measured the risks of acute urinary retention (AUR), BPH-related surgery, and urinary tract infection (UTI). The endpoints assessing the side effect profile of dutasteride included ED and gynecomastia.

**Table 1:** **Predictor variables included in each nomogram**.

	**PCa**	**HGPCa**	**ED**	**AUR**	**BPH**	**Gyn**	**UTI**	**HGPIN**	**ASAP**
Age	*	*	*	*	*	*	–	*	*
Prostate volume	*	*	–	*	*	–	–	*	*
No. of biopsy cores	*	*	–	–	–	–	–	*	*
PSA	*	*	–	*	*	–	–	*	*
% free PSA	*	*	–	–	–	–	–	*	*
Family history PCa	*	*	–	–	–	–	–	*	*
DRE	*	*	–	–	–	–	–	*	*
Body mass index	*	*	–	–	–	*	–	*	*
IPSS score	–	–	–	*	*	–	–	–	–
Qmax	–	–	–	*	*	–	*	–	–
Residual volume	–	–	–	*	*	–	*	–	–
Sexually active	–	–	*	–	–	–	–	–	–
History lack of libido	–	–	*	–	–	–	–	–	–
History impotence	–	–	*	–	–	–	–	–	–

PCa, prostate cancer; HGPCa, high-grade prostate cancer; ED, erectile dysfunction; AUR, acute urinary retention; BPH, benign prostatic hyperplasia; Gyn, gynecomastia; UTI, urinary tract infection; HGPIN, high-grade prostatic intraepithelial neoplasia; ASAP, atypical glands suspicious for carcinoma; DRE, digital rectal examination; IPSS, international prostate symptom score.

Predictive variables for each endpoint were selected by clinical relevance based on findings from the initial publication of the REDUCE trial (Table 1). Restricted cubic splines were implemented for continuous or ordinal variables to accommodate potential non-linear relationships. Multivariable analyses were then performed to measure the correlation between each variable and the outcome of interest.

For 7 of the 9 endpoints, Cox proportional hazards (PH) regression models were built from both patient sub-groups. For HGPIN and ASAP, two separate competing risks regression (CRR) models were used to investigate the cause-specific cumulative incidence of these endpoints. These Cox PH models and CRR models served as the basis of nomograms that would be used to predict each of the 9 outcomes. The discrimination of each model was quantified by calculating the concordance index (CI), which is identical to the non-parametric area under the receiver operating characteristic curve (AUC) in a binary setting, and modified to fit for time-to event or competing risks outcomes. All models were internally validated using resampling techniques: bootstrapping analysis with 1000 resamples for the Cox PH models and 10-fold cross-validation for the CRR models (to correct for over-fitting bias). In addition, cause-specific cumulative incidences of HGPIN or ASAP and probabilities of freedom from the other 7 endpoints were calculated for each of the models.

Calibration plots for each nomogram were plotted to measure how closely the predicted risk generated by the model approximated observed rates of the endpoint of interest. A prediction tool that is perfectly calibrated should demonstrate a 1:1 relationship between predicted and actual outcomes, resulting in a calibration plot with a 45° slope. Calibration was assessed visually by dividing patients into quartiles of the nomogram-predicted probabilities of freedom from event (or cumulative incidences of HGPIN or ASAP), and then plotting the mean predicted values against Kaplan-Meier estimated probabilities (or non-parametrically estimated cumulative incidences of HGPIN or ASAP) for each quartile.

All *p*-values were generated by two-sided statistical tests, with a level of 0.05 indicating significance. All statistical analyses were performed using R software version 2.11.0 (R Development Core Team, 2010) with the Design and cmprsk libraries added.

## Results

The characteristics of the placebo and dutasteride cohorts appeared to be comparable with no significant differences among any of the variables used as predictive markers in nomogram construction (Table 2). The results of the multivariable analyses assessing the predictive value of the clinical variables for each of the 9 endpoints for both the placebo and dutasteride cohorts are summarized in Table 3. It should be noted that some of the endpoints lacked any significantly predictive markers. For example, the variables of age and body mass index were not predictive of the risk of gynecomastia in either placebo or dutasteride groups on multivariable analysis. In the cases of BPH-related outcomes, markers that were significantly associated with the outcome in the placebo group (e.g., maximal urinary flow rate or prostate volume) were no longer predictive in the dutasteride group.

**Table 2:** **Patient characteristics**.

	**Placebo**	**Dutasteride**	***P*-value**
Number of patients	3424	3305	–
Age (years)	62.7	62.8	0.44272
Prostate volume (cc)	45.5	45.7	0.57712
Number of biopsy cores (mean)	8.7	8.8	0.37677
PSA (ng/dl)	5.9	5.9	0.75824
% free PSA	16.7	16.7	0.85107
BMI (kg/m^2^)	27.3	27.3	0.87295
IPSS score	8.5	8.6	0.31229
Qmax (mL/sec)	15.3	15.2	0.90313
Residual volume (mL)	46.2	47	0.49068
Family history of PCa			
*No*	2987	2853	0.32667
*Yes*	437	448	
DRE			
*Abnormal*	132	125	0.86863
*Normal*	3284	3176	
Ethnicity			
*Non-white*	295	277	0.73033
*White*	3129	3028	
Sexually active			
*No*	615	621	0.37376
*Yes*	2807	2680	
History of lack of libido			
*No*	2657	2569	0.8489
*Yes*	751	718	
History of impotence			
*No*	2505	2351	0.07281
*Yes*	902	934	

BMI, body mass index; IPSS, international prostate symptom score; Qmax, maximum flow rate; PCa, prostate cancer; DRE, digital rectal exam.

**Table 3:** **Hazard ratios and *P*-values from multivariate analysis**.

**Variables**	**Placebo**				**Dutasteride**			
	**HR**	**Lower 0.95**	**Upper 0.95**	***P*-value**	**HR**	**Lower 0.95**	**Upper 0.95**	***P*-value**
**Prostate cancer**								
Age	1.6175	1.4411	1.8155	<0.0001	1.5645	1.3712	1.7851	<0.0001
Prostate volume	0.7656	0.6917	0.8473	<0.0001	0.7343	0.6465	0.8341	<0.0001
No. of biopsy cores	0.8462	0.733	0.9769	0.0548^*^	0.8443	0.716	0.9954	0.004
PSA	1.1612	1.0283	1.3113	0.0088	1.1269	0.9837	1.291	0.1998
% free PSA	0.7177	0.6471	0.7958	<0.0001	0.7539	0.6721	0.8456	<0.0001
BMI	0.9932	0.902	1.0935	0.496	1.0504	0.9405	1.1731	0.3806
Family history PCa	1.5439	1.2824	1.8587	<0.0001	1.3815	1.117	1.7088	0.0029
DRE	1.2906	0.9184	1.8136	0.1417	1.0953	0.7269	1.6505	0.6634
**High grade prostate cancer**								
Age	2.6442	2.047	3.4155	<0.0001	2.1537	1.7027	2.7242	<0.0001
Prostate volume	0.6416	0.5329	0.7724	<0.0001	0.5777	0.4595	0.7262	<0.0001
No. of biopsy cores	0.7413	0.5654	0.9719	0.0952^*^	0.844	0.636	1.1201	0.1687
PSA	1.2826	1.0057	1.6358	0.067^*^	1.2544	0.9833	1.6002	0.1889
% free PSA	0.5645	0.4699	0.6781	<0.0001	0.5063	0.4175	0.614	<0.0001
BMI	1.0551	0.8776	1.2685	0.8195	1.0811	0.9053	1.2912	0.4994
Family history PCa	1.7416	1.2292	2.4676	0.0018	1.3653	0.9455	1.9714	0.0967
DRE	1.2037	0.6151	2.3558	0.5883	1.6935	0.9405	3.0493	0.0792
**Erectile dysfxn**								
Age	0.7339	0.6295	0.8557	0.0004	0.7621	0.666	0.8722	0.0001
Sexually active	0.5096	0.3675	0.7067	0.0001	0.3664	0.2681	0.5006	<0.0001
Hx lack of libido	1.3311	1.038	1.707	0.0242	1.3472	1.0819	1.6775	0.0077
Hx impotence	1.2409	0.9787	1.5734	0.0747	0.9972	0.8107	1.2265	0.9785
**Acute urianry retention**								
Age	0.8908	0.7196	1.1028	0.4819	0.8059	0.5185	1.2525	0.4953
Prostate volume	1.8827	1.4251	2.4873	<0.0001	0.9947	0.6244	1.5848	0.711
PSA	1.011	0.8024	1.2737	0.9935	1.2705	0.7303	2.2103	0.4152
IPSS	1.5953	1.1804	2.1558	0.0027	2.0617	1.0162	4.1826	0.0561^*^
Qmax	0.5879	0.472	0.7323	<0.0001	0.9401	0.5678	1.5566	0.7829
Residual volume	1.2595	0.9105	1.7424	0.3255	1.5552	0.74	3.2685	0.0976
**BPH-related surgery**								
Age	0.8127	0.6247	1.0572	0.0807	1.559	0.8902	2.7302	0.2985
Prostate volume	1.5691	1.1561	2.1297	0.0095	1.2977	0.7537	2.2343	0.6382
PSA	1.1172	0.8531	1.4629	0.2629	1.1834	0.6867	2.0396	0.6908
IPSS	2.5274	1.6817	3.7984	<0.0001	1.0011	0.5496	1.8234	0.0079^*^
Qmax	0.5939	0.4581	0.7701	0.0004	0.7832	0.4732	1.2961	0.5844
Residual volume	1.1945	0.8151	1.7505	0.623	0.9939	0.4937	2.0008	0.7463
**Gyn**								
Age	0.9581	0.6793	1.3514	0.9678	1.0843	0.8216	1.431	0.8477
BMI	1.3178	0.9245	1.87 82	0.2171	1.173	0.9042	1.5216	0.1553
**UTI**								
Qmax	0.7375	0.6153	0.8839	0.0023	1.0545	0.817	1.361	0.8655
Residual volume	1.243	0.9581	1.6125	0.2471	1.2778	0.9049	1.8044	0.2652
**HGPIN**								
Age	1.4293	1.1525	1.7725	0.0008	1.1009	0.8335	1.454	0.5191
Prostate volume	0.8821	0.7065	1.1015	0.5214	0.8965	0.6763	1.1884	0.746
No. of biopsy cores	1.1212	0.8356	1.5043	0.4359	0.7871	0.5426	1.1416	0.4507
PSA	0.9833	0.7943	1.2174	0.9196	1.1428	0.849	1.5383	0.667
% free PSA	1.1113	0.9045	1.3654	0.5567	1.1635	0.8752	1.5468	0.5078
BMI	0.9732	0.8194	1.1559	0.3665	1.1077	0.8449	1.4523	0.2359
Family history PCa	1.1008	0.7477	1.6206	0.6266	1.1135	0.6828	1.8161	0.6665
DRE	1.1732	0.6183	2.2262	0.625	1.5201	0.7052	3.2768	0.2852
**ASAP**								
Age	0.9748	0.786	1.2089	0.5177	1.0457	0.8091	1.3515	0.9228
Prostate volume	0.8217	0.6617	1.0203	0.0969	0.7806	0.6	1.0156	0.1721
No. of biopsy cores	1.0959	0.783	1.5337	0.7047	0.8367	0.6103	1.1471	0.0626
PSA	1.0578	0.8441	1.3255	0.8837	1.3181	0.9993	1.7387	0.1427
% free PSA	1.085	0.8719	1.3502	0.0485^*^	1.0896	0.8478	1.4005	0.4193
BMI	1.1344	0.9301	1.3835	0.3104	0.9405	0.7492	1.1808	0.7538
Family history PCa	1.1106	0.745	1.6554	0.6067	1.6653	1.1066	2.5061	0.0145
DRE	1.2115	0.6209	2.3639	0.5738	1.2553	0.5959	2.6445	0.5498

Gyn, gynecomastia; UTI, urinary tract infection, HGPIN, high grade prostatic intraepithelial neoplasia; ASAP, atypical small acinar proliferation.

* The use of restricted cubic splines may result in incongruency between significant p-values and hazard ratios that cross 1.0 as a result of the relaxed linearity assumptions.

The concordance indices for the component nomograms are summarized in Table 4. Several of the nomograms (e.g., those for UTI, gynecomastia, HGPIN, ASAP) demonstrate poor discrimination and are based on those models that contained a large proportion of non-predictive variables. Values of less than 0.5 reflect poor discrimination by a given nomogram and are an artifact of random assignment of risk scores to patients. If a greater number of cross-validations were to be run, the average predictive accuracy would likely be closer to 0.5. For the final metagram, these suboptimal nomograms were replaced by the overall cumulative incidence probabilities of the endpoint in question.

**Table 4:** **Nomogram concordance indices**.

**Outcomes**	**Placebo**	**Dutasteride**
PCa	0.61909	0.61205
HGPCa	0.6924	0.71333
ED	0.58557	0.59467
AUR	0.65849	0.61706
BPH-S	0.69534	0.59861
Gynecomastia	0.52166	0.52139
UTI	0.55017	0.51103
HGPIN	0.5321	0.47203
ASAP	0.48454	0.53215

PCa, prostate cancer; HGPCa, high grade prostate cancer; ED, erectile dysfunction; AUR, acute urinary retention; BPH-S, BPH related surgery; UTI, urinary tract infection; HGPIN, high grade prostatic intraepithelial neoplasia; ASAP, atypical small acinar proliferation.

Nomogram calibration appeared to correlate with how well the particular nomogram discriminated. The 4 nomogram pairs that predict UTI, gynecomastia, ASAP, and HGPIN demonstrated poor correlation between observed and expected outcomes, while those nomograms predicting any cancer, high grade cancer, ED, AUR, or BPH-related surgery demonstrated excellent calibration.

**Figure 1:**
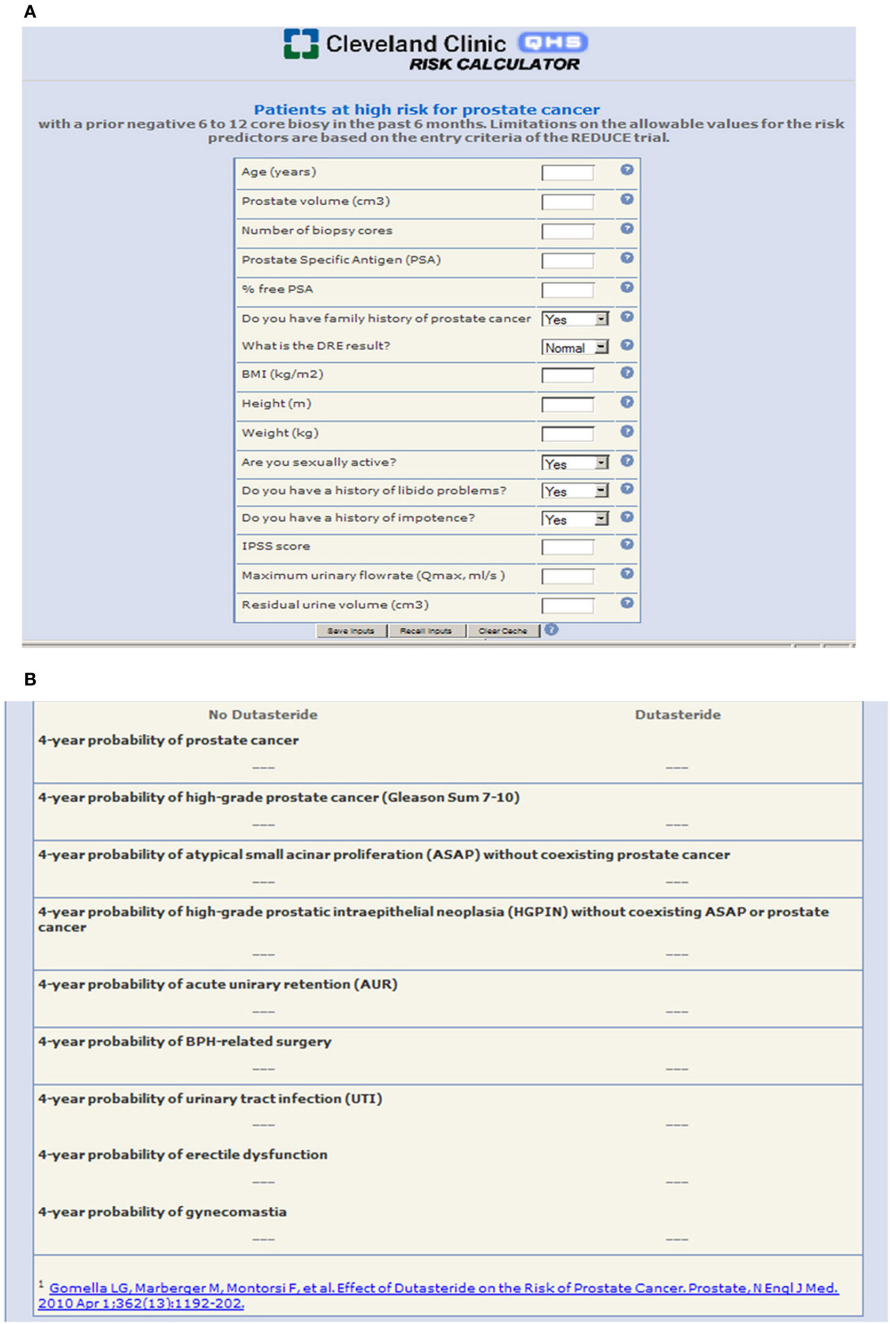
**(A)** The REDUCE metagram as a user-friendly online risk calculator with “plug and play” functionality where the patient or physician can input patient-specific variables to generate predictions; **(B)** Individualized patient outcomes are generated and presented in a clear and concise format.

The component nomograms were then incorporated into the final metagram, which has been made available as an online calculator (http://rcc.simpal.com/RCEval.cgi?RCID=eU9iCH) that can be used by physician or patient to generate personalized predictions of all 9 endpoints simultaneously (Figure 1).

## Discussion

Despite better understanding of its biology, improved screening tests, and availability of more efficacious therapy, PCa can still be associated with significant morbidity and mortality, particularly if found at an advanced stage. As such, there has been growing interest in modifying a man's risk of developing PCa through chemoprevention with drugs that alter the hormonal milieu of prostatic cells. Inhibitors of 5-alpha reductase, such as dutasteride, have been shown to reduce the risk of PCa by nearly 25% but are not without side effects. Consequently, assessing the balance between benefit and harm associated with dutasteride treatment is a critical aspect of counseling the patient considering chemoprevention.

In order to make informed decisions and reduce the risk of treatment regret, patients require unbiased, evidence-based data regarding probabilities of treatment success and complications. A formalized system that is easy to use and interpret and that can generate accurate tailored predictions can be useful to both patient and physician. We believe that a metagram, a comprehensive prediction software which incorporates highly accurate nomograms for each endpoint, is best suited to this purpose.

The REDUCE metagram can theoretically provide estimates of outcomes relevant to dutasteride treatment that are tailored to a man at risk for developing PCa (i.e., older men with elevated PSA and a history of previous negative biopsy). As an online risk calculator, our metagram can be used by a physician to enter patient-specific variables and generate a tabular presentation of personalized risk estimates. The patient can then make a truly informed decision regarding the appropriateness of chemoprevention based on the relative value he assigns to different outcomes and health states. For example, a man who values sexual function more than a chance of decreasing his cancer risk may decline dutasteride treatment if his metagram-predicted risk of ED is sufficiently high. On the other hand, a man who fears the development of cancer above all else may opt for chemoprevention even if his risk of PCa is minimal while his risks of side effects are high.

In its current state, there are certain limitations to the use of the REDUCE metagram in men considering chemoprevention. First, it should be noted that dutasteride is not FDA-approved for the indication of PCa risk reduction. This certainly does not preclude the use of 5-ARIs in general as chemopreventive agents, but patients must be adequately counseled regarding their on-label and off-label uses. Second, some of the nomograms, including those predicting the endpoints of HGPIN and ASAP, generated predictions that were comparable to random chance. This was related to the fact that those models completely lacked predictive markers in both the placebo and dutasteride cohorts. As a result, the overall cumulative incidence probabilities of those endpoints were used in place of nomogram-generated predictions. The application of group-level probabilities to the individual patient is problematic because the study group may not be representative of that particular patient. Furthermore, the nomograms for BPH-related outcomes (e.g., AUR, BPH-related surgery, and UTI) demonstrated reduced accuracy in the dutasteride cohort, likely due to modification of the value of baseline prostate-related markers by the drug itself. This shortcoming could be addressed by the construction of nomograms that incorporate post-treatment values for markers like urinary flow rate or prostate volume.

Third, the metagram does not predict for all potential adverse effects of dutasteride, namely the composite event termed “cardiac failure.” In the original study, investigators found a higher incidence of cardiac failure (which included conditions such as congestive heart failure, cardiac failure, acute cardiac failure, ventricular failure, cardiopulmonary failure, and congestive cardiomyopathy) among men who took dutasteride compared to placebo (0.7 vs 0.4%, *p* = 0.03) (Andriole et al., 2010). Because the original trial did not collect data on any clinical variables that correlate with cardiovascular status, we were unable to build a nomogram predicting cardiac failure and suggest that the cumulative incidence rates of this endpoint from the REDUCE trial be used to counsel patients and supplement the metagram-generated predictions.

It should be noted that even among the nomograms that demonstrated predictive accuracies greater than random chance, none predicted with 100% accuracy. As such, there is opportunity to improve the predictive performance of the metagram by improving its component nomograms. This can be achieved through utilization of larger datasets, identification and incorporation of better predictive markers, standardization of data collection methodology, and use of more sophisticated modeling techniques.

Taken together, these considerations emphasize that nomogram predictions must be interpreted as such; they are not perfect and may not be applicable to all men at risk for PCa. By themselves, nomograms cannot make treatment recommendations nor can they take the place of patient counseling. The current role of prediction models, like the REDUCE metagram, in clinical practice is to provide patients with the best estimates of their relevant individual outcomes, which, combined with physician judgment and patient preference, can then form the basis for truly informed decision-making regarding the utility of dutasteride chemoprevention.

Using data from the REDUCE trial, we have created a comprehensive prediction tool that can simultaneously predict the potential benefits and adverse effects of dutasteride treatment and help determine the appropriateness of chemoprevention for men at high risk for PCa. The metagram, in its current state, does not predict all relevant outcomes with adequate accuracy but does provide the framework for future research into the indications and consequences of treatment with 5-ARIs.

### Conflict of interest statement

The authors declare that the research was conducted in the absence of any commercial or financial relationships that could be construed as a potential conflict of interest.
